# A nursing perspective on leadership within the National Health Service

**DOI:** 10.1177/17449871251403353

**Published:** 2026-01-20

**Authors:** Emily Palmer

**Affiliations:** Adult Nurse, Oxford Brookes University, Oxford, UK

*‘*“*Let whoever is in charge keep this simple question in her head: not, how can I always do this right thing myself, but how can I provide for this right thing to always be done?’ –* Florence Nightingale

## Understanding nursing leadership

Leadership has always been integral to nursing practice in the United Kingdom (UK). From the first professional schools established under Florence Nightingale to contemporary National Health Service (NHS) roles, nurses have balanced advocacy, coordination, and decision-making in complex clinical environments. However, leadership in nursing is most often understood within the confines of the ward or service level, focused on team management, patient safety, and quality improvement (Cummings et al., 2021). These forms of leadership are essential, but they are not the only leadership spaces where nurses can make a meaningful contribution.

The wider structures of health and care, integrated care boards, commissioning bodies, and system partnerships, shape the environment in which care is delivered. Decisions taken at these levels influence workforce planning, digital innovation, service development, and ultimately patient outcomes. Yet nurses are often absent from these discussions. The introduction of general management into the NHS in the 1980s separated the worlds of clinical practice and management (Ham, 2020; Maxwell, 2023). Medical professionals retained defined routes into system leadership, but nursing’s organisational voice became fragmented and operational rather than strategic.

The NHS Graduate Management Training Scheme (GMTS) has long served as a structured pathway for developing future leaders in healthcare management. It is, however, a scheme primarily designed for non-clinical entrants. Engaging with the programme as a registered nurse provided a unique lens through which to observe how leadership is cultivated in non-clinical contexts, and to consider how similar developmental opportunities might be better tailored for clinicians. At present, nurses who aspire to influence organisational or policy decision-making often have to navigate routes designed for general management, as structured equivalents in clinical leadership remain comparatively limited (Storey and Holti, 2013; West et al., 2015).

## The boundaries of leadership

Leadership theory offers a foundation for understanding why nursing voices matter in organisational contexts. Transformational leadership theory emphasises the role of vision, motivation, and empowerment in achieving shared goals (Bass, 1990). This aligns with nursing’s relational and values-based approach to care, which seeks to influence through empathy, credibility, and example rather than authority (Cummings et al., 2021). Similarly, frameworks of shared governance promote distributed leadership and collaborative decision-making, recognising that high-quality care arises from collective accountability rather than hierarchical control (Hess, 2011).

Clinical systems leadership has emerged as a particularly relevant concept for the current NHS context (Stanley, 2014; West et al., 2015). It positions clinicians as integrators who connect operational experiences with strategic objectives, ensuring that system decisions reflect the lived experience of staff and patients. This form of leadership depends on nurses being confident and credible within multidisciplinary and organisational settings, yet traditional leadership training often fails to prepare nurses for these roles.

Historically, the underrepresentation of nursing voices in organisational leadership is not due to a lack of capability, but rather the absence of formal mechanisms to enable it. Research from The King’s Fund (West et al., 2020) and NHS Leadership Academy (2018) highlights that nurses frequently lead complex change at ward level but are seldom provided with access to strategic forums. This creates a ‘translation gap’ between the realities of patient care and the policies intended to improve it.

## Connecting clinical and system perspectives

Placements with an Integrated Care Board, a management consultancy, and a modern district general hospital provided insight into how strategic priorities are formed, interpreted, and implemented across the system. Each environment revealed different perspectives on leadership and performance. Commissioning emphasised governance and policy alignment; consultancy valued analytical rigour and efficiency, and the hospital context highlighted patient experience and operational pressure.

A time and motion study exploring elective caesarean sections demonstrated how qualitative contextual data could enrich the understanding of patient flow and staff experience. The ability to interpret both quantitative and qualitative findings through a clinical lens helped to illuminate inefficiencies and relational dynamics that might otherwise be overlooked. This integration of system-level metrics with clinical insight provided a more holistic view of performance, one grounded in both human experience and organisational design.

Throughout these experiences, the absence of clinical voices in system discussions was striking. Nursing perspectives tended to appear by coincidence rather than by design. When present, they added crucial understanding of how strategies were enacted on the ground, how change affected staff behaviour, and how patients experienced care pathways. The ability to connect policy to practice in this way emphasises a central strength of nursing leadership: its capacity to bridge the organisational and the clinical, ensuring that system decisions remain anchored on the patient experience.

## Professional presence

The professionalisation of NHS management in the 1980s, following the Griffiths Report, created a managerial identity largely separate from clinical professions (Ham, 2020). This structural separation has had lasting implications. Nursing leadership, once a visible part of hospital administration, became increasingly confined to operational management roles. The resulting gap between clinical and managerial spheres has limited nursing influence at policy level and contributed to a perception that strategic leadership belongs primarily to non-clinicians (Maxwell, 2023; West et al., 2020).

This imbalance is problematic not only for professional equity but also for system effectiveness. Policies developed without clinical insight risk overlooking how reforms play out in practice (West et al., 2015). Workforce initiatives designed purely around efficiency can undermine staff morale or continuity of care if they fail to consider the relational aspects of nursing work. Similarly, digital transformation programmes can struggle with implementation when frontline realities are insufficiently understood (NHS England, 2023).

Nurses are uniquely positioned to counter these challenges. Their grounding in patient-centred care provides a vital corrective to policy abstraction. Clinical systems leadership encourages nurses to engage confidently with data, finance and governance while maintaining a focus on human experience (Cummings et al., 2021; Stanley, 2014). For this to be realised, however, organisational structures must make space for nurses to lead beyond traditional boundaries. This requires intentional representation in decision-making forums, mentorship from experienced clinical leaders and leadership development programmes that bridge rather than separate management and practice.

## Nursing insight in organisational leadership

The case for integrating nursing insight into organisational and policy leadership is not only ethical but also strategic. Studies repeatedly show that organisations with strong nursing leadership deliver better patient outcomes, higher staff satisfaction, and improved safety culture (West et al., 2020; Wong et al., 2013). These findings underline that nursing leadership is not peripheral to system success; it is fundamental to it.

Developing nurses who can operate confidently across clinical and managerial domains demands structured exposure to system-level decision-making. Programmes such as the GMTS can offer valuable insight, but the experience of clinical participants highlights a broader issue: that equivalent, accessible leadership pathways for nurses, and other clinicians, remain scarce. Initiatives such as the NHS Leadership Academy’s Clinical Leadership Programme and the Chief Nursing Officer’s strategic fellowships are important steps forward, yet appear later in a clinician’s career and are less visible than general management routes.

Leadership development for nurses should be grounded in nursing theory and professional identity. Transformational leadership and shared governance frameworks provide models for inclusive and values-based leadership (Bass, 1990; Hess, 2011). These principles align closely with the UK Nursing and Midwifery Council’s (NMC, 2018) emphasis on advocacy, person-centred care, and professional integrity. Embedding these concepts into system leadership programmes would help ensure that policy and strategy reflect not only operational targets but also the human experience at the heart of care.

## A stronger nursing voice in management

Nursing has always been central to patient care, but its influence at strategic and policy levels remains uneven. The health challenges facing the NHS today: workforce sustainability, digital transformation, and integrated care, demand leadership that is both clinically informed and system-aware.

Early-career nurses bring fresh insight grounded in clinical experience and professional values. When supported to engage in system leadership, they can act as translators between practice and policy. The experience of navigating a management training route designed for non-clinical staff illustrates both the value of cross-professional exposure and the gap in opportunities for clinicians to develop equivalent expertise. Addressing this imbalance is essential if nursing is to fulfil its potential as a leading voice in healthcare transformation.

Strong nursing leadership is not an optional enhancement to system governance; it is a prerequisite for compassionate, effective, and equitable healthcare. By integrating nursing perspectives into organisational and policy decision-making, the NHS can ensure that strategic priorities remain focused on the true lived experience of patients and clinicians.

Nightingale’s words, ‘*Let whoever is in charge keep this simple question in her head - not, how can I always do this right thing myself, but how can I provide for this right thing to be always done?*’, remain profoundly relevant. They capture the essence of leadership as stewardship: not about control or individual achievement but about creating the conditions in which good care can consistently happen.

That principle underpins every aspect of nursing leadership, whether at the bedside or in strategic conversations. It is the responsibility and privilege of nurse leaders to ensure that compassion, evidence, and the patient voice are not confined to the clinical setting, but are embedded in the very systems that shape how care is delivered. In this way, Nightingale’s challenge continues to guide us to lead not only by doing but also by enabling the right thing to be done.

As the NHS continues to evolve, the voice of nursing leadership will remain essential is shaping compassionate, sustainable, and patient-centred care for the future.
